# A new technique combining virtual simulation and methylene blue staining for the localization of small peripheral pulmonary lesions

**DOI:** 10.1186/1471-2407-14-79

**Published:** 2014-02-11

**Authors:** Yang Shentu, Liang Zhang, Hengle Gu, Feng Mao, Minghui Cai, Zhengping Ding, Zhiqiang Wang

**Affiliations:** 1 Department of Thoracic Surgery, Shanghai Chest Hospital, Shanghai Jiao Tong University, Shanghai 200030, China; 2 Department of Thoracic Oncology Medicine, Jilin Tumor Hospital, Changchun 130012, Jilin Province, China; 3 School of Medicine, University of Queensland, QLD 4029 Queensland, Australia

**Keywords:** Lung cancer, Pulmonary lesions, Thoracoscopy, Localization, Simulation, Methylene blue

## Abstract

**Background:**

Quickly and accurately localizing small peripheral pulmonary lesions can avoid prolonged operative time and unplanned open thoracotomy. In this study, we aimed to introduce and evaluate a new technique combining virtual simulation and methylene blue staining for the localization of small peripheral pulmonary lesions.

**Methods:**

Seventy four (74) patients with 80 peripheral pulmonary lesions <20 mm in size on computer tomography (CT) were virtually punctured using a radiotherapy planning simulator on the day before operation. Under general anaesthesia, methylene blue dye was injected to the virtually identified point according to the surface point, angle and depth previously determined by the simulator. The wedge resection of the marked lesion was performed under video-assisted thoracoscopic surgery (VATS) and the specimens were sent for immediate pathologic examination. According to pathology results, appropriate surgical procedures were decided and undertaken.

**Results:**

The average lesion size was 10.4±3.5 mm (range: 4-17 mm) and the average distance to the pleural surface was 9.4±4.9 mm. Our preoperative localization procedure was successful in 75 of 80 (94%) lesions. Histological examination showed 28 benign lesions and 52 lung cancers. The shortest distance between the edges of the stain and lesion was 5.1±3.1 mm. Localization time was 17.4±2.3 min. All patients with malignant lesions subsequently underwent lobectomy and systematic lymph node dissection. No complications were observed in all participants.

**Conclusions:**

The novel technique combining the preoperative virtual simulation and methylene blue staining techniques has a high success rate for localizing small peripheral pulmonary lesions, particularly for those tiny lesions which are difficult to visualise and palpate during VATS.

## Background

In recent years, the widespread utilization of CT scanning and the increasing awareness of health screening have resulted in identification of a large number of small pulmonary lesions. It has been reported that a large number, about 59% to 73%, of those lesions with a localized area of ground glass opacity nodules are early stage cancers [1] while some lesions are benign. Generally, the wedge resection of the nodule is performed first for pathologic examination, and further treatment planning for small peripheral pulmonary nodules will depend on pathologic findings to prevent unwanted thoracotomy. However, the challenge that we often face during the surgery is to quickly and accurately localize those small nodules, particularly those tiny nodules which are difficult to be quickly palpated. Inadequate nodule localization might lead to a prolonged operative time and even conversion to an unplanned open thoracotomy [2,3]. Therefore, several preoperative localization techniques have been introduced as a method of improving the success rate of video-assisted thoracoscopic surgery (VATS) [4-9]. Each technique has its strengths and limitations. In this study, we introduce a novel technique which applies the virtual simulation, a technique for radiotherapy planning [10,11], in combination with methylene blue staining for the localization of small peripheral pulmonary nodules. In this study, we describe this procedure and assess the performance of this technique in 80 small peripheral pulmonary lesions from 74 Chinese patients.

## Methods

### Participants

From February 2012 to February 2013, 74 participants aged from 29 to 81 years were recruited from Shanghai Chest Hospital for medical check-ups. All participants met the following criteria. First, they underwent chest CT scanning with an identified peripheral pulmonary lesion <20 mm in diameter in the outer third of the lung field. Those lesions were not directly visible on the lung surface. Second, those lesions had not been pathologically examined before this study so that the diagnosis of the lesions was unknown. Third, no other overt abnormalities were detected in blood routine, blood glucose, electrolyte and ECG results. They had normal lung, liver, kidney and blood coagulation functions. Abdominal ultrasound, brain MRI, and whole body bone scan showed no signs of metastases in those patients. Participants with at least one of the following were excluded: 1) lesions >20 mm which could be easily localized, 2) lesions located in the middle and inner two thirds of the lung field where the wedge resection was not feasible, 3) lesions close to the pleural surface with visible pleural retraction, 4) impossible to inject methylene blue dye because the identified lesions were blocked by a large skeletal structure such as scapula according to CT scanning results, and 5) lesions close to heart, large blood vessels, diaphragm and major nerves with a high risk of injuring them. Among 74 participants recruited, 80 lesions were identified. As the probability of finding malignancy among the participants with lesions was high, all eligible participants with lesions went through the proposed localization procedure. This project was approved by the Ethics Review Committee of Shanghai Chest Hospital and informed consent was obtained from all participants, including consent for participation, and consent to publish findings and to use anonymous images in medical publications.

### Virtual simulation ─ methylene blue staining technique

The virtual simulation ─ methylene blue staining technique contains preoperative and intraoperative localization procedures.

#### Preoperative localization

**Figure 1:**
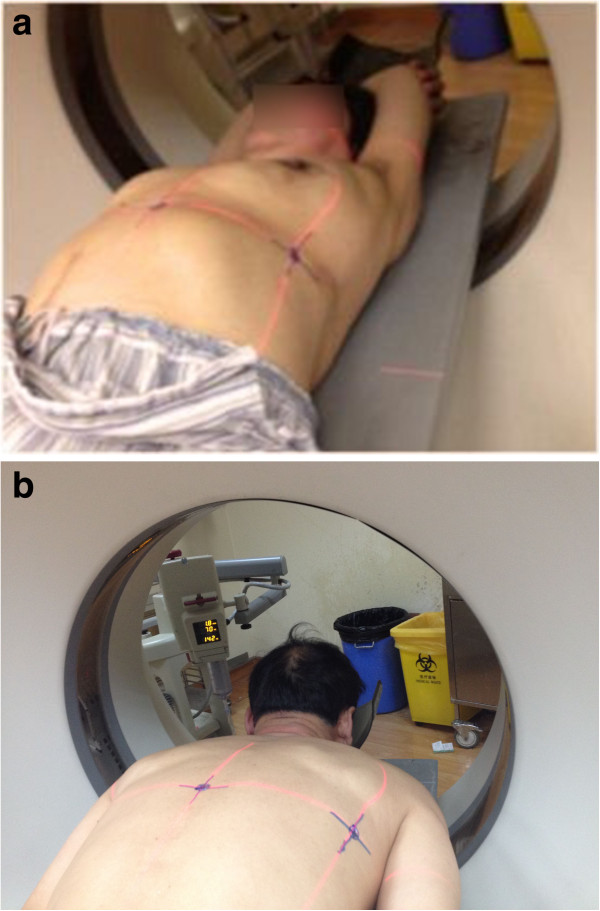
**CT Scanning to collect data for simulation.** Spine **(a)** and **(b)** prone position.

**Figure 2:**
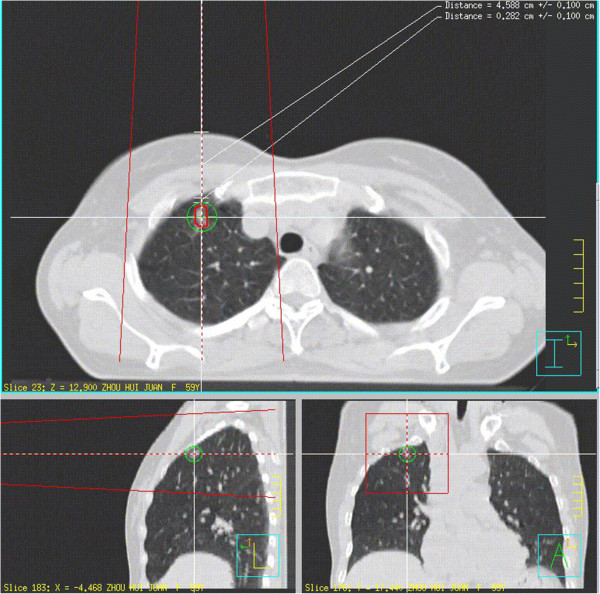
Image generated using radiation therapy planning system based on images and data collected from previous CT scan.

**Figure 3:**
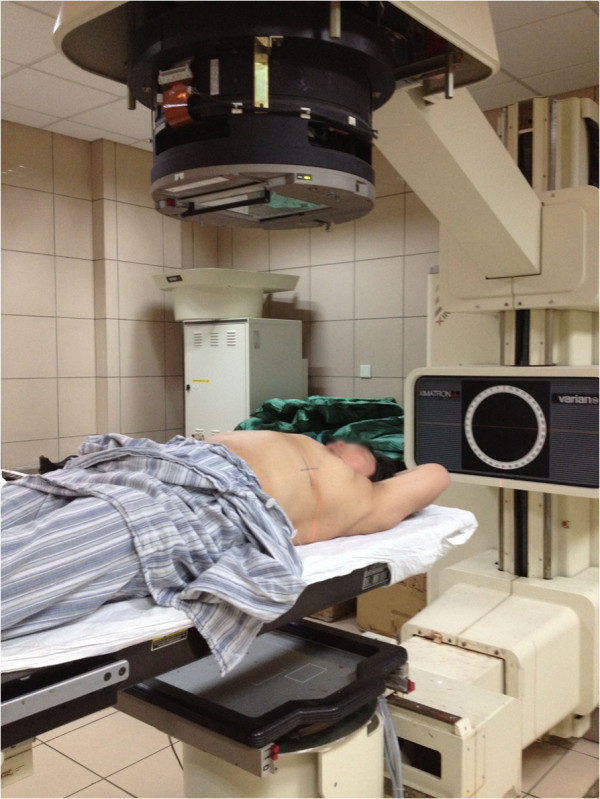
Patient on a simulator for radiotherapy planning in the same position as that for the original CT scan to perform a full 3 D simulation.

**Figure 4:**
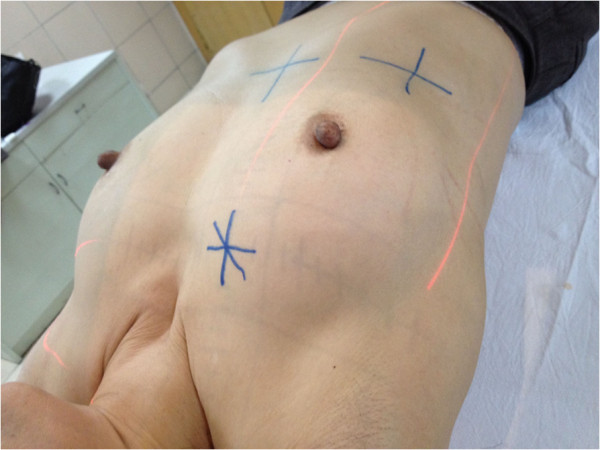
Identifying and marking the surface puncture point.

**Figure 5:**
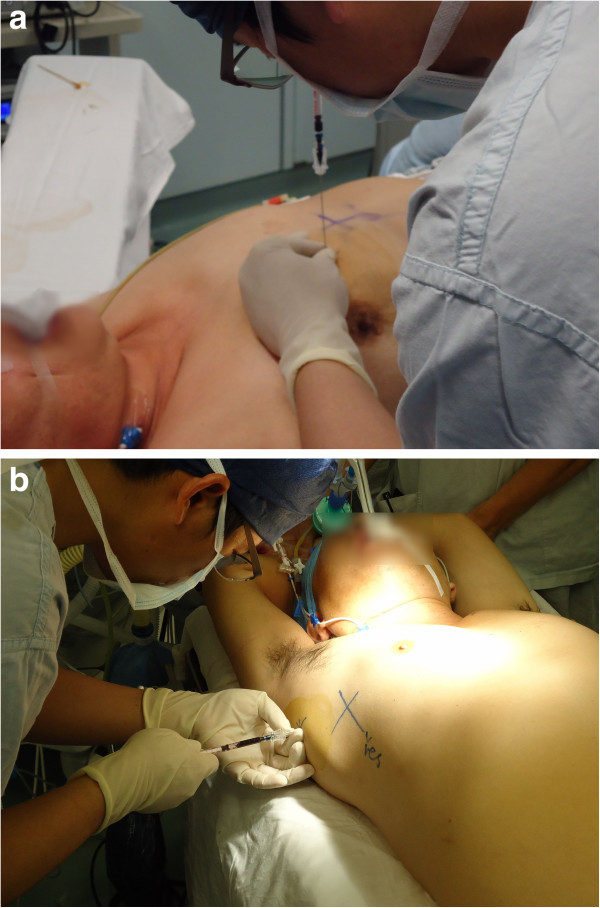
**Injection of methylene blue dye to the virtually identified point under general anaesthesia. a)** Spine position with vertical injection. **b)** Spine position with horizontal injection.

1) CT scanning: On the day before the operation, a CT scan was performed in the Department of Radiology at the Shanghai Chest Hospital using Siemens SOMATOM Sensation 16 (Siemens AG, Medical Solutions, Germany). Accompanied by one researcher (LZ), patients were placed on the CT table in either a spine or prone position which allowed the shortest access to the targeted lesion. The spine position (Figure 1a) was suitable for injecting methylene blue dye from the front or axilla area while the prone position (Figure 1b) was for injecting from the back. The targeted lesion was located with several scans of contiguous 2 mm section thickness in full inspiration. The researcher marked the alignment lines of the CT gantry with a “+” sign, placing a metallic mark.

2) Image transferring and simulation data for virtual puncturing: The CT images generated in the Department of Radiology were transferred to the Department of Radiotherapy in our hospital, in which an injection plan was developed using the radiation therapy planning system, Phillips-Pinncle 3 (Philips Healthcare). This system was originally designed for developing radiation therapy and we used the various parameters generated from this system for a different purpose: localizing lesions. According to the CT images, the system software calculated the direction and depth of the radiation beam to the lesion. It also calculated the distance between skin surface to the lesion and the distance from the edge of the lesion to the pleural surface. For our purpose, we envisaged that the injection needle could act as a beam and follow the direction of the beam to reach the targeted lesion according to the distance from skin surface to the lesion calculated by the system. For each lesion, the system provided various entry points and angles. We chose the point with either vertical or horizontal angle to minimize operational difficulties and errors (Figure 2). The simulated virtual data for puncturing were recorded for the next step.

3) Puncture point marking: After obtaining the above data, we placed the patient on a simulator (Ximatron series) for radiotherapy planning in the same position as that for the original CT scan to perform a full 3 D simulation. To ensure the same position as that when the data were collected previously, the alignment lines of the CT gantry aimed at those three “+” signs. Guided by those simulation data, through the computer controlled machine and bed movements, the puncture point was identified and marked as “*” (Figure 3 and Figure 4).

4) Injecting methylene blue in the operation room: Under general anaesthesia, the patient was placed in the same position as that for preoperative CT image collection, and 0.3 ml methylene blue was injected at the marked point according to the angle and depth data and again another 0.2 ml at 10 mm from pleural during needle removal to stain the needle pathway (Figure 5).

#### Intraoperative localization procedures

**Figure 6:**
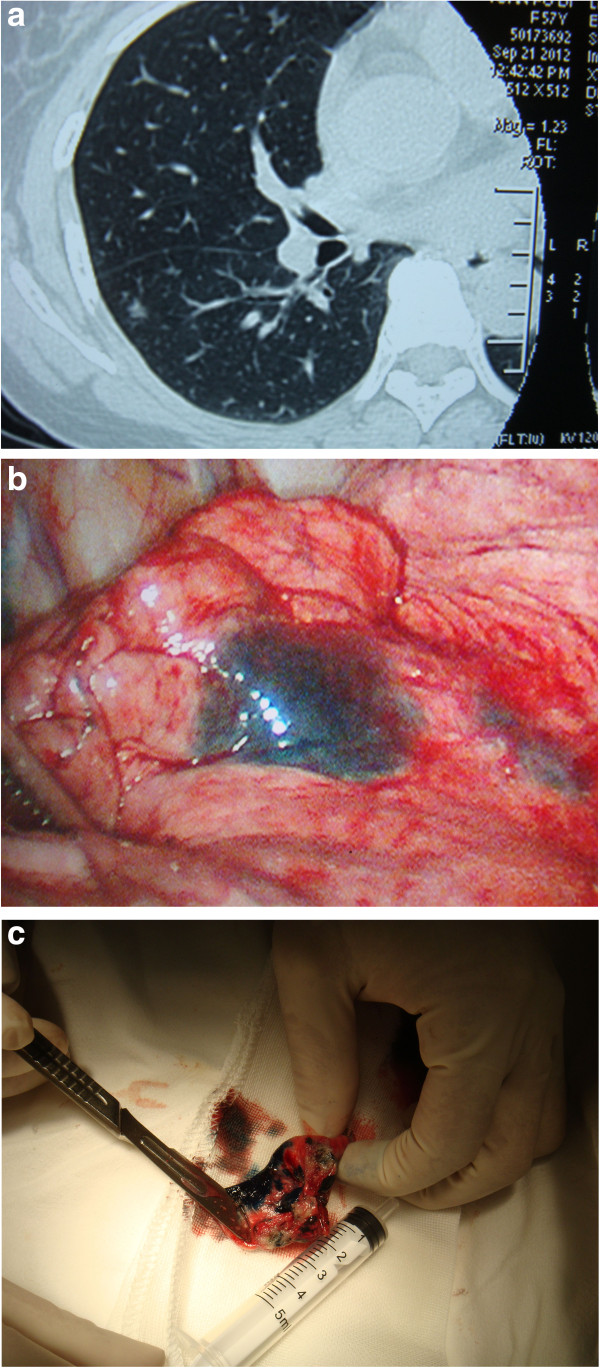
**A lesion on right lower lobe. a)** CT Image, **b)** Thoracoscopic view, and **c)** Specimen of wedge resection.

Immediately after injecting methylene blue, the skin was disinfected and thoracoscopy was performed to locate the stain. The surrounding surface was palpated using the index finger for the lesion. The wedge resection of the identified lesion was performed using *VATS* along with a 3 cm margin of normal lung tissue, including the stained area, and the specimen was placed next to a 5 ml syringe which was taken as a reference length scale to measure the distance between the edge of the lesion and the edge of the stain (Figure 6). The specimen was sent for immediate pathologic examination. All patients with malignant lesions subsequently underwent lobectomy and systematic lymph node dissection. For those with benign lesions, the surgery was completed.

### Measurements and data analysis

The preoperative localization time was defined as the period from the time start of CT scanning to the time of marking skin puncture point. Intra-operative localization time was from the injection of methylene blue to the time that the stain was located with the thoracoscope. The success of the localization was determined according to the distance between the edge of the stain and that of the lesion (<20 mm). The success proportions and 95% confidence interval were calculated. Linear regression models were used to assess if age, gender, and the location, distance from pleural surface and size of lesions were associated with the accuracy of this technique. All analyses were performed with Stata SE 12 [12].

## Results

### Characteristics of participants and lesions

Table 1 shows the characteristics of 74 study participants (27 men and 47 women) and 80 lesions. About 31% study participants were smokers. Two cases had previously been diagnosed with a malignant tumor: one underwent surgery for rectal cancer two years after the identification of the current lesion and the other had been received surgery for thyroid cancer three years before. The mean lesion size was 10.4 mm (SD: 3.5), ranging from 4 mm to 17 mm. The mean intra-operative localization time was 17.4 (SD: 2.3) minutes, ranging from 12 to 23 minutes.

**Table 1:** Characteristics of 74 study participants and 80 peripheral pulmonary lesions

**Characteristics**	**Mean (SD)**
Age, years	55.1 (10.7)
Male, n (%)	35 (37.5)
Smoker, n (%)	23 (31.1)
History of prior malignancy	2 (2.7)
Family history of malignancy	3 (4.1)
Diameter, mm	10.4 (3.5)
Distance from lesion to the pleural surface, mm	9.4 (4.9)
Preoperative localization time, minutes	22.2 (5.0)
Intraoperative localization time, minutes	17.4 (2.3)
Stain – lesion distance, mm	5.1 (3.1)

### Performance of virtual simulation ─ methylene blue staining technique

Seventy five of 80 lesions were successfully localized and the successful localization rate was 94% (95% CI: 86, 98). The stain-lesion distance for those successfully localized lesions was 5.3 (SD: 3.1), ranging from 0 to 12 mm. Histological examination showed 28 benign lesions and 52 lung cancers.

**Figure 7:**
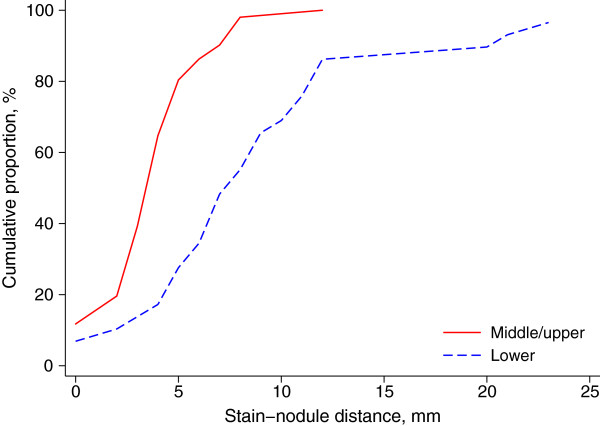
Cumulative distributions of stain-lesion distance for lesions on middle/upper versus those on lower lobes.

Table 2 shows the stain-lesion distances and successful localization rates by location and pathological diagnosis. There was no significant difference in stain-lesion distance between left and right lungs. The stain-lesion distances were not dependent on the type of histological diagnosis, lesion sizes, age and sex. The lower lobes had a significantly longer stain-lesion distance than upper and middle lobes with a crude average of 3.0 (95% CI: 1.6, 4.3) mm (p<0.001). Further controlling for age, sex, lesion size and pathological diagnosis using the multiple linear regression method, the adjusted difference between lower and upper/middle lobes in stain-lesion distance remained statistically significant with a mean of 3.0 (95% CI: 1.7, 4.4), p<0.001. However, about 80% stain-lesion distances were<5 mm for lesions on middle/upper lobes while 80% stain-lesion distances were <10 mm for lesions on the lower lobes (Figure 7). The successful localization rate was 98% (95% CI: 90, 100) for lesions in upper or middle lobes, which was significantly higher than that for lesions in lower lobes (86%), p=0.036. There were no significant differences in the successful localization rate or stain-lesion difference between left and right lungs and between benign and malignant lesions.

**Table 2:** Stain – nodule distance and successful localization rates by different characteristics

	**N**	**Stain – lesion distance (mm)**		**Successful localization, %**	
		**Mean (SD)**	**P**	**Rate (95% CI)**	**P**
Left vs right lobes					
Left	28	5.1 (2.9)		93 (76, 99)	
Right	52	5.0 (3.2)		0.90	94 (84, 99)	0.81
Upper/middle vs lower						
Upper/Middle	51	4.1 (2.4)			98 (90, 100)	
Lower	29	7.0 (3.4)		<0.001	86 (68, 96)	0.036
Diagnosis						
Benign	28	5.4 (2.8)			96 (82, 100)	
Malignant	52	4.9 (3.3)		0.46	92 (81, 98)	0.47
Lesion size						
≤10 mm	42	4.9 (2.7)			93 (81, 99)	
>10 mm	38	5.2 (3.5)		0.76	95 (82, 99)	0.73
Lesion to pleural distance, mm						
<10 mm	39	5.1 (2.8)			95 (83, 99)	
≥10 mm	41	5.0 (3.4)		0.82	93 (80, 98)	0.69
Age						
<55 years	35	5.1 (3.0)			95 (82, 99)	
≥55 years	40	5.0 (3.2)		0.87	93 (81, 99)	0.77
Sex						
Male	29	5.9 (2.6)		97 (83, 100)		
Female	46	4.6 (3.3)		0.074	92 (81, 98)	0.20

All patients with successfully localized malignant lesions subsequently underwent lobectomy via VATS and systematic lymph node dissection. The postoperative pathology examination revealed all cancer cases as stage T1aN0M0. Wedge resection was performed to remove benign lesions. There were no complications observed in any of the patients as a result of the localization. Among five unsuccessful localized lesions, four were later localized through thorough index palpation around 20-23 mm from the stain. Only one lesion was not localized and the patient underwent thoracotomy. Since the pathologic examination confirmed as a lung cancer case, this patient underwent lobectomy by thoracotomy.

## Discussion

In this study, we demonstrated that a new technique could be used to successfully localize small peripheral pulmonary lesions. We found this technique performed well regardless of the pathological diagnosis, lesion size, and distance from lesion to the pleural surface, and patient’s age and gender. This technique performed significantly better for the lesions in upper lobes than in lower lobes. This technique combines the existing virtual simulation technique for planning radiotherapy and methylene blue staining.

With the increasing application of CT to lung cancer screening, small lesions are frequently detected [13,14]. However, for small lesions, localizing them visually or by palpation during VATS can be difficult [5,6]. To shorten the time for nodule localization and to prevent unplanned open thoracotomy, several preoperative localization techniques have been introduced as a method of improving the success rate of VATS. Punkett et al. developed a technique of hookwire localization with CT guidance [15-17]. This approach has a high successful localization rate, but increases the risk of some complications including pneumothorax, pleuritic pain, and haemorrhage [18]. It is also associated with a relatively high level of radiation for both patients and doctors. The VATS should be performed immediately after the hookwire placement, which may cause operational difficulties in a busy hospital like ours. Lenglinger et al. developed the percutaneous staining with methylene blue method [8]. Guided by CT, methylene blue dye is injected into the nodule. This method also has a high successful localization rate and no complications associated with the previously mentioned hookwire placement [8,19,20]. However, the operation should be performed immediately after the injection before the dye is diffused. To overcome this problem, Nomori and Horio developed a long-lasting point marker termed “colored collagen” which combining atelocollagen, methylene blue and contrast medium [21]. The colored collagen stayed visible at the injected site for several days without toxicity. Another marking technique called “agar marking” was developed by Tsuchida et al. [22]. Powdered agar dissolved in distilled water and kept at >50 degree Celsius to maintain its liquid form which is injected to the target lesion guided by CT. Then, agar can be detected as a hard nodule by palpation, but this approach is difficult to implement. Shennib et al. reported the use of intraoperative intrathoracic ultrasonography for localization of small lesions during VATS [23], which has been assessed by others as a non-invasive and easy to operate technique [24,25]. However, this technique does not perform well for small lesions <10 mm, particularly for ground glass pulmonary nodules. It also does not perform well for patients with asthma or chronic obstructive pulmonary disease because of the influence of air in the lung tissue. Recently, Chen et al. reported an image-guided navigation system for localization of small pulmonary nodules before thoracoscopic surgery [26]. Although this technique can successfully localize the nodules without some shortcomings of other techniques, the system is costly and complex to operate and has not been commonly used in the current clinical settings.

In our study, we introduced a new technique combining the existing virtual simulation for radiotherapy planning technique and methylene blue staining for the localization of small peripheral pulmonary lesions. With a successful localization rate of 94%, this technique has the following advantages. First, the doctors are not exposed to radiation during the preoperative localization period, and the patients are only exposed to radiation during the initial CT image collection. Second, the patients do not need to be sent to the operating room immediately after virtual puncture, which allows sufficient time for preoperative preparation. Third, patients are less stressed when methylene blue dye was injected under general anaesthesia. Forth, since the injection is performed under general anaesthesia, complications such as pneumothorax and haemorrhage are rare and can be relatively easily handled. Fifth, the operation is performed immediately after the methylene blue injection with little time for the dye to diffuse around the tissue. Finally, since the virtual puncture can be performed using equipment commonly available, it can be easily adopted by most hospitals without extra cost for new equipment. The special equipment required for this technique is a CT simulator (radiation therapy planning system), which is generally available in most hospitals with facilities for radiation therapy. The cost of methylene blue dye is minimal and the total cost of the whole procedure is equivalent to that of a chest CT scan.

However, there are some limitations. First, unlike the real-time CT guided localization methods, the accuracy of our technique depends on the similarity between the body position for virtual puncture and that for injecting the dye. Any inconsistency may cause deviation from the targeted lesion, particularly for the cases in which injection needs to be performed from the back. At the early stage of the study, we instructed patients to cross their hands on the back of the head in a prone position during virtual puncture. Such a position is difficult to repeat during the injection when patients are under general anaesthesia. To minimize such deviation, during the simulation puncture, we instructed patients to naturally rest their hands to their thighs to imitate a position as the one when they were under general anaesthesia for the dye injection. Second, if the injection path is blocked by a costa, the needle entry point should be moved upward or downward and the needle angle will also be changed. This may also cause an inaccurate staining point. Our data showed that the technique performed much better for lesions in the upper and middle lobes than those in the lower lobes. The poor performance for localizing lesions in the lower lobe could be partly explained by those limitations. Our technique failed to localize five of the 80 lesions, three of them were due to inconsistent body positions between simulation and actual injection as previously described, one was due to the lesion being close to the pleural surface and one due to too much dye being injected causing wide diffusion around the tissue. Further investigation is needed to improve the performance of this technique for localizing small peripheral lesions, particularly those in the lower lobes.

From our clinical experience of successful localization, it is important to keep the same body position for the dye injection as that during the simulation. If possible, a horizontal or vertical injection angle should be adopted to inject approximately 0.3 ml of methylene blue dye into the target lesion site and again about 0.2 ml into about 10 mm from the pleural surface during the removal of the needle. Too much dye should be avoided to prevent a large stained area.

## Conclusions

Applying CT simulation technique to virtually localize small peripheral lesions and injecting methylene blue dye to the target point according to the simulated data can lead to a high successful localization rate. This technique does not require new equipment and the patients have lower exposure to radiation compared to using a real-time CT guided approach. Injection of the methylene blue dye under general anaesthesia minimises patients stress and associated complications. This technique is safe, effective and readily acceptable with an overall successful localization rate of 94%, even higher for those lesions in middle and upper lobes (98%). Further efforts are needed to improve the successful localization rate and minimize errors.

## Abbreviations

CT: Computer tomography; VATS: Video-assisted thoracoscopic surgery.

## Competing interests

The authors declare that they have no competing interests.

## Authors’ contributions

This study was conceived and designed by YS and LZ. YS, LZ, HG, FM, MC and ZD participated in the acquisition of data. YS, LZ and ZW participated in the statistical analysis, drafting and critical revision of the manuscript. All authors read and approved the final manuscript.

## Pre-publication history

The pre-publication history for this paper can be accessed here:

http://www.biomedcentral.com/1471-2407/14/79/prepub
